# SARS-CoV-2 Omicron subvariants exhibit distinct fusogenicity, but similar sensitivity, to pan-CoV fusion inhibitors

**DOI:** 10.1080/22221751.2023.2178241

**Published:** 2023-02-23

**Authors:** Shuai Xia, Lijue Wang, Fanke Jiao, Xueying Yu, Wei Xu, Ziqi Huang, Xicheng Li, Qian Wang, Yun Zhu, Qiuhong Man, Shibo Jiang, Lu Lu

**Affiliations:** a Key Laboratory of Medical Molecular Virology (MOE/NHC/CAMS), Shanghai Institute of Infectious Disease and Biosecurity, School of Basic Medical Sciences, Shanghai Frontiers Science Center of Pathogenic Microbes and Infection, Fudan University, Shanghai, People’s Republic of China; b Department of Clinical Laboratory, Shanghai Fourth People's Hospital, School of Medicine, Tongji University, Shanghai, People’s Republic of China; c National Laboratory of Biomacromolecules, Institute of Biophysics, Chinese Academy of Sciences, Beijing, People’s Republic of China

**Keywords:** SARS-CoV-2, Omicron subvariants, fusogenicity, cell–cell fusion, fusion inhibitor

## Abstract

Continuous emergence of the Omicron variant, along with its subvariants, has caused an increasing number of infections, reinfections, and vaccine-breakthrough infections, seriously threatening human health. Recently, several new Omicron subvariants, such as BA.5, BA.2.75, BA.4.6, and BF.7, bearing distinct mutation profiles in their spike (S) proteins, have significantly increased their capacity to evade vaccine-induced immunity and have shown enhanced infectivity and transmissibility, quickly becoming dominant sublineages. In this study, we found the S proteins of these Omicron subvariants to have 2- to 4-fold more efficient membrane fusion kinetics than that of the original Omicron variant (BA.1), indicating that these novel Omicron subvariants might possess increased pathogenicity. We also identified that peptide-based pan-CoV fusion inhibitors, EK1 and EK1C4, showed equal efficacy against membrane fusion mediated by S proteins of the noted Omicron subvariants and infection by their pseudoviruses. Additionally, either immune sera induced by wild-type (WT) SARS-CoV-2 RBD-based vaccine or BA.2 convalescent sera showed potent synergism with EK1 against both WT SARS-CoV-2 and various Omicron subvariants, further suggesting that EK1-based fusion inhibitors are promising candidates for development as clinical antiviral agents against the currently circulating Omicron subvariants.

## Introduction

In late 2021, the Omicron variant B.1.1.529/BA.1 was first identified in South Africa and quickly spread to many countries [1,2]. It exhibited distinct immunogenicity from that of wild-type (WT) SARS-CoV-2 or other SARS-CoV-2 variants of concern (VOCs), and it successfully escaped from immunity induced by first-generation COVID-19 vaccines [3,4]. Subsequently, this variant quickly became the globally dominant SARS-CoV-2 variant [1]. However, the Omicron variant (BA.1) has continuously expanded into hundreds of subvariants, or sublineages, including BA.2, BA.2.12.1, BA.4, and BA.5, which showed enhanced transmissibilty and quickly overtook the ancestral Omicron subvariant BA.1 [4]. Most recently, several new Omicron subvariants have emerged, particularly BA.2.75, BA.4.6, and BF.7, also exhibiting increased transmissibility, resulting in a large number of reinfections and causing global concern [5–7].

Different Omicron subvariants contain distinct mutation profiles, especially in their S proteins, well known to play a crucial role in mediating viral infection. Compared with WT SARS-CoV-2 S protein, Omicron BA.1 contains more than 30 spike mutations. In particular, K417N, N440K, G446S, S477N, T478K, E484A, Q493R, G496S, Q498R, N501Y, and Y505H mutations are known to occur in the Omicron BA.1 receptor binding domain (RBD) (Figure S1). Such mutations significantly alter the conformation of RBD in S protein and, hence, seriously threaten clinical vaccine efficacy [4]. Compared with BA.1, BA.2 contains some unique spike mutations, such as T376A, D405N, and R408S (Figure S1), similar to the special L452R and F486V RBD mutations in BA.4/BA.5 S protein, which have significantly enhanced their ability to evade immune surveillance [8,9]. For example, numerous studies reported that BA.4 and BA.5 subvariants reinfected those patients who had already recovered from BA.1 or BA.2 infections [9,10]. BA.2.12.1 evolved from BA.2 with L452Q RBD mutation, and BA.2.75 evolved with G446S and N460K RBD mutations. Additionally, BA.2.75 contains many unique mutations in the spike N-terminal domain, such as K147E, W152R, F157L, I210V, and G257S (Figure S1). BA.4.6 and BF.7 subvariants were originated from BA.4 and BA.5 subvariants, respectively. BA.4.6 spike protein contains the additional R346T and N658S mutations, while only R346T occurred in BF.7 S protein. The epidemic-causing advantages of these novel Omicron subvariants call for clarification of their specific spike-mediated virological characteristics in order to develop effective antiviral agents.

Previous studies reported that S-mediated fusogenicity plays an important role in viral pathogenesis [11]. For example, the Delta variant exhibited increased fusogenicity, compared with that of wild-type SARS-CoV-2, accompanied by significantly enhanced pathogenicity *in vivo* [12]. On the contrary, although the transmissibility of Omicron BA.1 surpasses that of WT SARS-CoV-2 or its other previous VOCs, the fusogenicity of Omicron BA.1 is significantly reduced, consistent with its decreased clinical pathogenicity [13]. Nevertheless, we shall see below that S-mediated fusogenicity should always be carefully monitored during Omicron variant evolution.

After receptor engagement, heptad repeat 1 (HR1) and 2 (HR2) regions in the S2 subunit of coronavirus S protein interact to form a six-helix bundle (6-HB) structure, which, in turn, drives viral fusion with and entry into the host cell [14]. Our previous study showed that HR1 is a conserved target against which we successfully developed a pan-CoV fusion inhibitor, EK1 [15]. The protype peptide of EK1, HR2P, was derived from the HR2 region of HCoV-OC43, and EK1 peptide was shown to broadly and effectively inhibit infection of all HCoVs tested, including SARS-CoV-2 [16]. By adding cholesterol (Chol) and “GSGSG-PEG4” linker to the C-terminus of EK1, the lipopeptide EK1C4 exhibited significantly increased antiviral potency over that of EK1 [16].

In the current study, we found that the recently emerging Omicron subvariants showed strengthened fusion kinetics, particularly BA.2.75, BA.4.6, BA.5, and BF.7, compared to that of BA.1, indicating that they possess increased pathogenicity. Although these circulating Omicron subvariants carry distinct mutation profiles in S protein, their HR1 functional domains remain conserved. Accordingly, we found that our EK1-based fusion inhibitors [15–17] maintained potency against these novel Omicron subvariants based on the results of S protein-meditated cell–cell fusion assay and pseudovirus infection. More importantly, together with either WT SARS-CoV-2 RBD-immunized mouse sera or BA.2 convalescent sera, EK1 showed strong synergisitc antiviral effect against infection by multiple Omicron subvariants. This finding suggest that EK1-based fusion inhibitors may exhibit enhanced efficacy against the current Omicron subvariants in vaccinees or COVID-19 convalescents.

## Results and discussion

### Distinct S-mediated fusogenicity among various omicron subvariants

**Figure 1.**
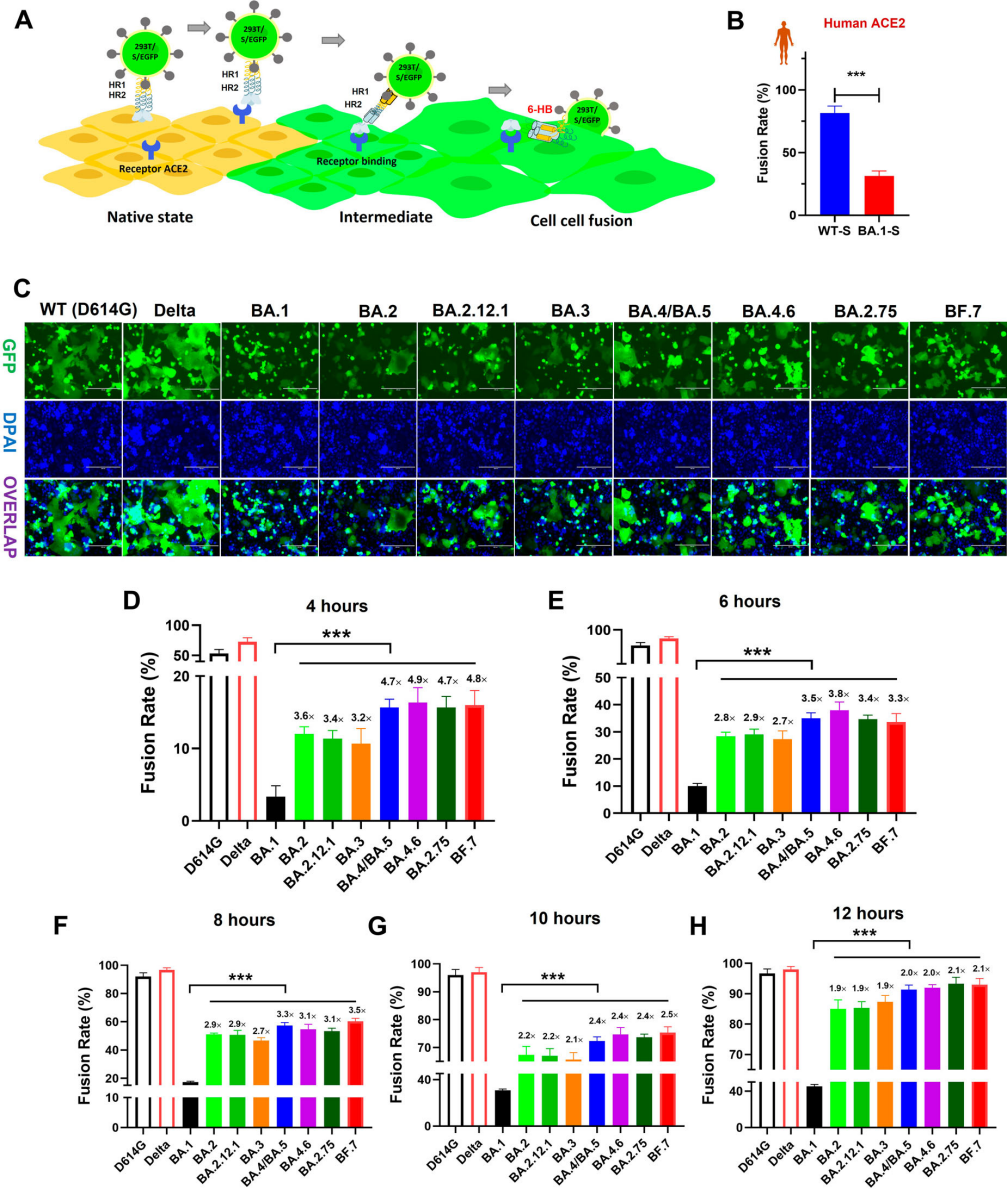
Decreased fusogenicity of Omicron subvariants. (a), Schematic representation of SARS-CoV-2 S-mediated cell-cell fusion. (b), Fusion rate mediated by WT and Omicron-BA.1 S proteins on HeLa cells expressing human-ACE2 after coculture for 10 h. (c), Representative images of S protein-mediated cell-cell fusion between 293T/WT(D614G)-S, Delta-S, BA.1-S, BA.2-S, BA.2.12.1-S, BA.3-S, BA.4/BA.5-S, BA.4.6-S, BA.2.75-S or BF.7-S /EGFP cells (effector cells) and Caco2 cells (target cells) after coculture for 12 hrs. Scale bar = 400 µm. (d-h), Fusion rate mediated by WT(D614G)-S, Delta-S, BA.1-S, BA.2-S, BA.2.12.1-S, BA.3-S, BA.4/BA.5-S, BA.4.6-S, BA.2.75-S and BF.7-S proteins on Caco2 cells after coculture for 4 (d), 6 (e), 8 (f), 10 (g) and 12 (h) hours. Samples were tested in triplicate, and the experiment was repeated once. Data from a representative experiment are presented in mean ± SD. Asterisks indicate significant differences (***P < 0.001).

We first evaluated the fusogenicity of different Omicron subvariants. To mimic authentic viral fusion with target cells, as mediated by S protein, their S proteins were expressed on the membrane surface of 293T-GFP cells, as effector cells (293T-S-GFP) (Figure 1(A)). Compared to WT-S, the capacity to mediate membrane fusion of BA.1 S protein was remarkably reduced on HeLa cells expressing human ACE2 (Figure 1(B)), consistent with previous results [18,19]. Other than human ACE2, it is well known that SARS-CoV-2 can also engage ACE2 receptors derived from various animals to drive viral infection [20]. Here, we evaluated whether mutant BA.1-S protein could also engage animal ACE2 derived from horse, cattle, swine, rabbit, and civet, or bat with either increased or decreased fusogenicity. On HeLa cells bearing these individual animal-ACE2 receptors, respectively, BA.1-S mediated membrane fusion in a manner similar to that of WT-S, but with significantly decreased fusogenicity (Figure S2). This line of evidence suggests that BA.1 may effectively infect some related animals, but with limited pathogenicity. Therefore, it can be concluded that the decreased fusogenicity property of ancestral Omicron variant BA.1 might ubiquitously exist in human and other animal hosts.

Meanwhile, we also used human intestine-derived cell line Caco2, naturally expressing human ACE2 receptor, as target cells to evaluate S-mediated fusogenicity of these Omicron subvariants. As expected, effector cells bearing either WT-S or Delta-S protein could fuse with target Caco2 cells to form larger fused cells containing multiple nuclei after coincubation for 12 h (Figure 1(C)). However, consistent with the above results, BA.1-S could only mediate limited fusion. On the other hand, we found that S proteins of BA.2, BA.2.12.1, BA.3, BA.4/BA.5, BA.2.75, BA4.6, and BF.7 all showed significant recovery in their fusion-mediating capability and drove more syncytium formation on Caco2 cells, compared to BA.1-S (Figure 1(C)).

Next, we systematically assessed the fusion kinetics mediated by the same S proteins at multiple time points (Figure 1(D–H) and Figure S3). As shown in Figure 1(D), significant differences in S-mediated fusion were observed among WT (D614G) SARS-CoV-2, Delta variant and Omicron subvariants after coincubation for 4 h. While WT-S and Delta-S proteins were driven by 53% and 73% fusion, respectively, BA.1-S was, again, driven by very limited fusion kinetics (about 3%). Interestingly, at the 4-hour time point, BA.2, BA.2.12, and BA.3 drove 12%, 11.3%, and 10.7% fusion, respectively, about 3 times that of BA.1-S-mediated fusion (Figure 1(D)). Moreover, BA.4/BA.5, BA.2.75, BA4.6, and BF.7 S protein drove 15.7%, 16.3%, 15.7%, and 16% fusion, respectively, rates more than 4-fold that in the BA.1-S group (Figure 1(D)).

At the 6-hour time point, the fusion rate driven by WT-S and Delta-S proteins was above 80%, whereas BA.1-S only drove about 10% fusion. BA.2-S-, BA.2.12-S-, and BA.3-S-mediated fusion was 28.3%, 29%, and 27.3%, respectively, rates 2.7- to 2.9-fold over that of BA.1-S-mediated fusion (Figure 1(E)). At the same time, BA.4/BA.5, BA.2.75, BA4.6, and BF.7 S protein fusion rates were 35%, 38%, 34.7% and 33.7%, respectively (Figure 1(E)). At the 8-hour time point post-coculture, WT-S and Delta-S mediated about 95% fusion, which is basically saturated, while BA.1-S only drove 17.3% fusion. In contrast to BA.1-S, BA.2-S, BA.2.12-S, and BA.3-S mediated 46.7% to 51% fusion, and BA.4/BA.5, BA.2.75, BA4.6, and BF.7 drove fusion ranging from 53.3% to 60.3% (Figure 1(F)). Similarly, after an additional 2 h, the rate of BA.1-S-mediated fusion was slowly increased to 30.7%, respectively. However, BA.2-S-, BA.2.12.1-S- and BA.3-S-mediated fusion still showed significant strengthening with 65.7% to 67.3% fusion, while the BA.4/BA.5, BA.2.75, BA4.6, and BF.7 groups showed 72.3% to 75.3% fusion (Figure 1(G)). Finally, at the 12-hour time point, the fusion rate of the BA.1-S group was 45.3%, but the S-mediated fusion of other Omicron subvariants showed more than 85% fusion, which tends to be saturated, just as the fusion meditated by WT-S or Delta-S (Figure 1(H)). In general, therefore, compared with BA.1-S, S proteins of all other tested Omicron subvariants showed more efficient fusion dynamics with significant difference, especially BA.4/BA.5, BA.2.75, BA4.6, and BF.7, suggesting that the strength of viral fusogenicity might be an important evolutionary direction for Omicron subvariants.

### Efficacy of EK1-based pan-CoV fusion inhibitors against Omicron subvariant S-mediated cell–cell fusion

Considering the trend toward increased fusogenicity of these novel Omicron subvariants, we asked if HR1-based fusion inhibitors could maintain their efficacy, as previously demonstrated against SARS-CoV-2 and its VOCs, including Alpha, Beta, Gamma, and Delta [21]. Although these variants contain numerous mutations in their S proteins, their S2 subunits are relatively conserved, especially in HR1 regions (Figure S4(A)), which are the target of fusion inhibitors [22]. Among these Omicron subvariants, only three HR1-mutations have been found, including Q954H, N969K and L981F, none of which seems to affect the major interface of EK1-HR1 [23] (Figure 4S(A,B)). Therefore, we herein further evaluated the efficacy of EK1 and EK1C4 against the fusion process mediated by S proteins of these Omicron subvariants.

**Figure 2.**
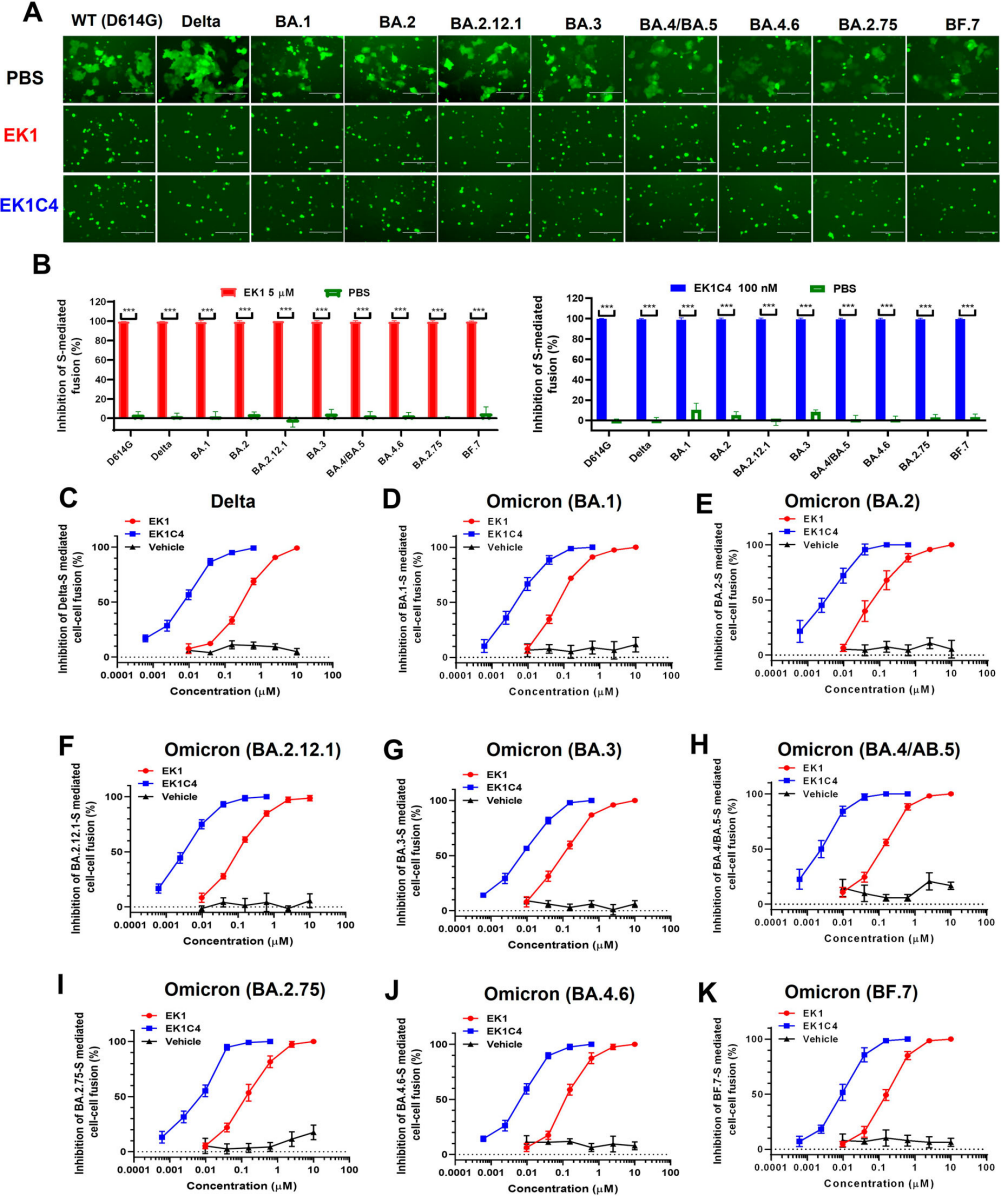
Efficacy of EK1 and EK1C4 against cell-cell fusion mediated by S proteins of SARS-CoV-2 and its variants. (a), Representative images of cell-cell fusion mediated by WT(D614G)-S, Delta-S, BA.1-S, BA.2-S, BA.2.12.1-S, BA.3-S, BA.4/BA.5-S, BA.4.6-S, BA.2.75-S and BF.7-S on Caco2 cells after coculture for 10 h in the presence of EK1 (5 µM), EK1C4 (0.1 µM) or PBS; scale bar = 400 µm. (b), Inhibition of EK1 (5 µM) and EK1C4 (0.1 µM) on S-mediated cell-cell fusion. (c-k), Efficacy of EK1 and EK1C4 against Delta-S-, BA.1-S-, BA.2-S-, BA.2.12.1-S-, BA.3-S-, BA.4/BA.5-S-, BA.2.75-S-, BA.4.6-S-, and BF.7-S-mediated cell-cell fusion. Samples were tested in triplicate, and the experiment was repeated once. Data from a representative experiment are presented in mean ± SD. Asterisks indicate significant differences (***P < 0.001).

As shown in Figure 2(A,B), in the presence of either EK1 (5 µM) or EK1C4 (0.1 µM), membrane fusion mediated by WT-S, Delta-S or BA.1-S was completely blocked. Meanwhile, numerous syncytia had formed by target cells and effector cells in the PBS group after coculture for 10 h. Both EK1 (5 µM) and EK1C4 (0.1 µM) also completely inhibited BA.2-S-, BA.2.12.1-S-, BA.3-S-, BA.4/BA.5-S-, BA.4.6-S-, BA.2.75-S-, and BF.7-S-mediated cell–cell fusion (Figure 2(A,B)).

Consistent with previous results, as alluded to above, here EK1 showed potent fusion inhibitory activity against WT-S-, Delta-S- and BA.1-S-mediated cell–cell fusion with concentrations for 50% inhibition (IC50s) of 142.02, 172.45 and 65.84 nM, respectively, while EK1C4 showed even more efficacy with IC50s ranging from 3.67 nM to 5.07 nM (Figure 2(C,D), Figure 5S(A) and Table 1). On BA.2-S-, BA.2.12.1-S-, BA.3-S-, or BA.4/BA.5-S-mediated cell–cell fusion, both EK1 and EK1C4 showed highly effective inhibitory activity with IC50s in the range of 72.37–85.89 nM and 2.31–4.38 nM, respectively (Figure 2(E–H) and Table 1). Moreover, EK1 and EK1C4 retained their broad and potent fusion inhibitory activity against these recent Omicron subvariants. For example, either EK1 or EK1C4 potently inhibited BA.2.75-S-mediated fusion with IC50 values of 89.07 and 4.20 nM, respectively (Figure 2(I) and Table 1). BA.4.6-S-mediated fusion process was also very sensitive to EK1 and EK1C4, showing IC50 values of 98.90 and 5.46 nM, respectively (Figure 2(J) and Table 1). Similarly, EK1 and EK1C4 significantly blocked BF.7-S-mediated cell–cell fusion with IC50 values of 93.76 and 3.76 nM, respectively (Figure 2(K) and Table 1).

**Table 1.** Inhibitory activity of EK1 and EK1C4 against SARS-CoV-2 subvariant S-mediated cell-cell fusion and pseudovirus (PsV) infection.

SARS-CoV-2	EK1 (IC50, nM)		EK1C4 (IC50, nM)	
	PsV	Cell-cell fusion	PsV	Cell-cell fusion
WT (D614G)	589.45 ± 130.62	142.02 ± 43.83	13.69 ± 4.61	4.48 ± 0.38
Delta	293.93 ± 153.27	174.25 ± 34.34	9.87 ± 1.75	5.07 ± 0.68
BA.1	300.44 ± 51.36	65.84 ± 8.36	5.28 ± 0.24	3.67 ± 0.64
BA.2	255.98 ± 71.39	77.09 ± 17.52	2.20 ± 0.33	2.31 ± 0.85
BA.2.12.1	409.36 ± 69.53	85.89 ± 26.77	7.95 ± 0.58	3.00 ± 0.60
BA.3	299.49 ± 92.81	72.37 ± 10.61	11.17 ± 9.46	3.18 ± 0.56
BA.4/BA.5	372.86 ± 51.20	84.45 ± 12.59	7.81 ± 2.53	4.38 ± 0.75
BA.2.75	245.58 ± 85.61	89.07 ± 22.92	6.21 ± 0.45	4.20 ± 0.59
BA.4.6	538.76 ± 268.54	98.90 ± 30.30	4.19 ± 0.36	5.46 ± 0.77
BF.7	213.39 ± 67.99	93.76 ± 29.18	5.62 ± 1.40	3.76 ± 1.27

### Potency of EK1-based pan-CoV fusion inhibitors against infection by pseudotyped Omicron variant and its subvariants

**Figure 3.**
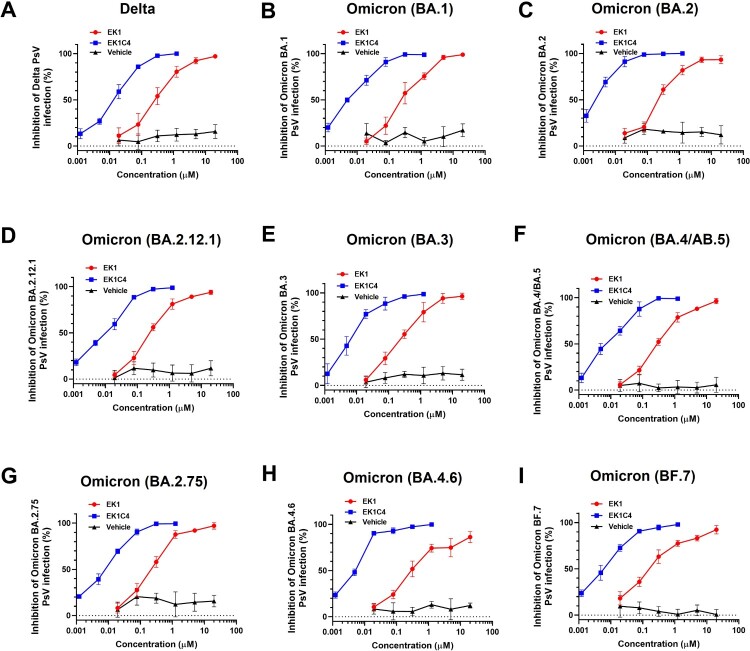
Efficacy of EK1 and EK1C4 against infection of pseudotyped SARS-CoV-2 and its variants. (a-k), Efficacy of EK1 and EK1C4 against Delta (a), BA.1 (b), BA.2 (c), BA.2.12.1 (d), BA.3 (e), BA.4/BA.5 (f), BA.2.75 (g), BA.4.6 (h) and BF.7 (i) PsV infection. Samples were tested in triplicate, and the experiment was repeated once. Data from a representative experiment are presented in mean ± SD.

To further assess the efficacy of EK1-based pan-CoV fusion inhibitors against Omicron subvariants, we developed lentivirus-based nonreplicative pseudovirus systems for these Omicron subvariants. Such constructs can effectively mimic the entry process of authentic virus and are, therefore, widely used to evaluate antiviral agents [24]. Both EK1 and EK1C4 showed potent inhibitory activity against WT, Delta and BA.1 infection (Figure 3(A,B), Figure S5(B) and Table 1), consistent with our previous results [25]. For BA.2, BA.2.12.1, BA.3, and BA.4/BA.5 subvariants, EK1 and EK1C4 showed potent efficacy with IC50s ranging from 255.98–409.36 nM or from 2.20–11.17 nM, respectively, (Figure 3(C–F) and Table 1). BA.2.75 is originated from BA.2 with several additional mutations in its S protein, which significantly escaped from BA.2-related immunity in recovered patients [5,26]. Nevertheless, EK1 and EK1C4 effectively blocked BA.2.75 infection with IC50 of 245.58 and 6.21 nM, respectively, (Figure 3(G) and Table 1). The sharp rise in infection cases has been caused by BA4.6 and BF.7 derived from BA.4/ BA.5 [25]. Nonetheless, EK1 could effectively inhibit both BA4.6 and BF.7 infection with IC50 values at 538.77 and 213.39 nM, respectively, and EK1C4 showed inhibition against BA4.6 and BF.7 with IC50s ranging from 4.19–5.62 nM (Figure 3(H,I) and Table 1). In general, then, both EK1 and EK1C4 maintained potent inhibitory activity against all tested Omicron subvariants, which is consistent with the above cell–cell fusion results, further attesting to the advantage of pan-CoV fusion inhibitors against the current Omicron epidemic. Although EK1 is now in clinical trials, its short half-life and lack of oral bioavailability may limit future clinical application. Most recently, however, we have identified a novel cyclic γ-AApeptide, S-20-1, which has potent fusion inhibitory activity against SARS-CoV-2 and its variants, as well as MERS-CoV, SARS-CoV, HCoV-OC43, HCoV-229E, and HCoV-NL63. It also exhibits long half-life (t_1/2_: 24h) and potential oral bioavailability by targeting SARS-CoV-2 HR1 in S2 subunit and RBD in S1 subunit [27]. At the same time, we have designed and engineered an HR2-targeting recombinant protein, 5-Helix, which consists of 3 HR1 and 2HR2 fragments. It interacts with the HR2 in viral S2 subunit to block homologous 6-HB formation between viral HR1 and HR2 domains, thus inhibiting infection by SARS-CoV-2 and its variants [28]. These findings suggest that either S-20-1 or 5-Helix could be further developed for therapeutic or prophylactic use, either alone or in combination with EK1, to treat or prevent infection by SARS-CoV-2 and its variants. Additionally, several other SARS-CoV-2 HR1-targeting inhibitors, such as HY3000 and YKYY017 that may be derived from P3 peptide [29] and IPB24 peptide [30], respectively, are being developed in clinical or preclinical trials.

### Potent synergism of EK1 combined with WT-RBD-immunized mouse sera or BA2-convalescent sera against omicron subvariants

**Figure 4.**
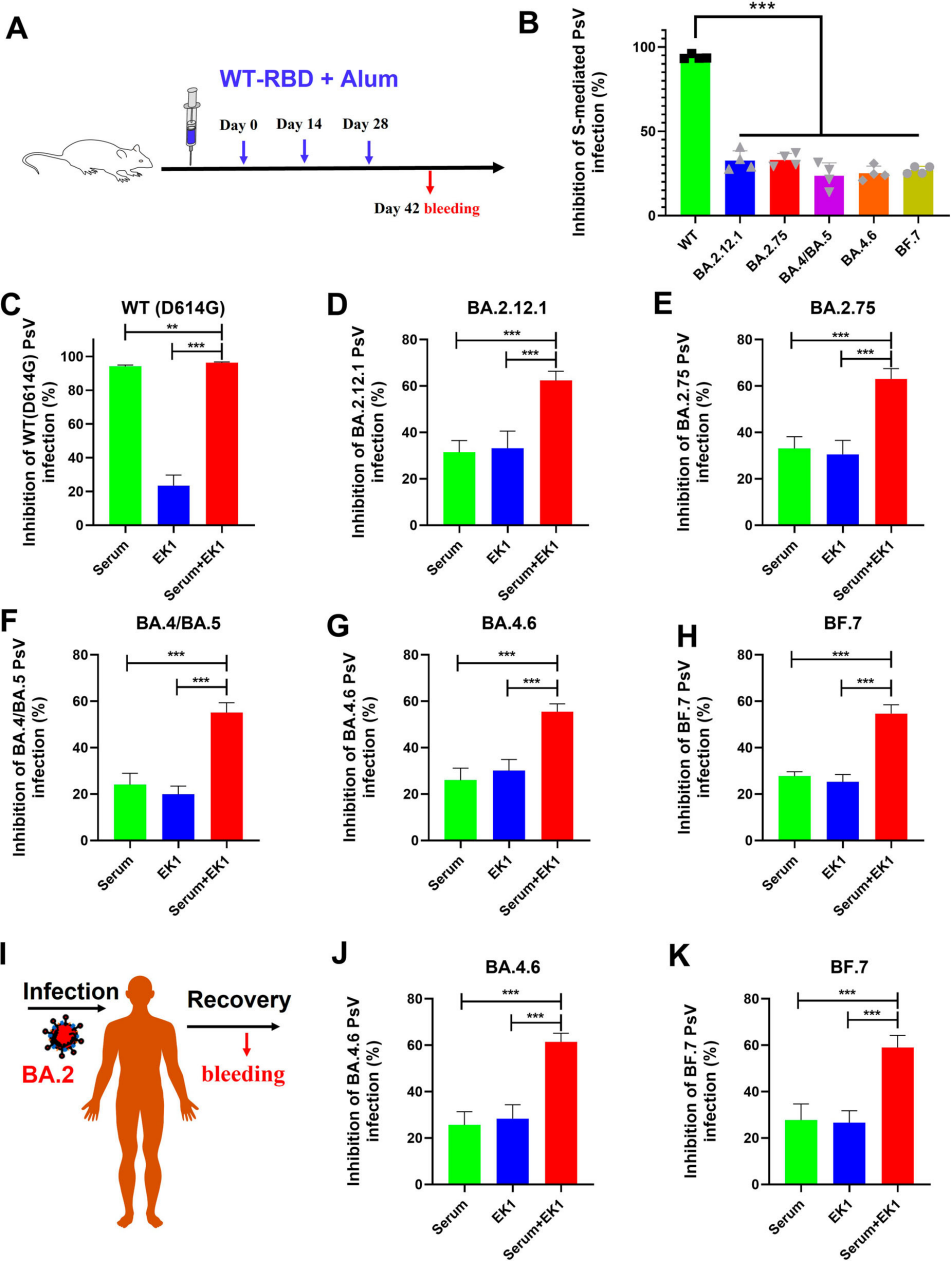
Potent synergism of EK1 combined with WT-RBD-immunized mouse sera or BA.2-convalescent sera against Omicron subvariants. (a) WT-RBD immunization protocols. (b) Efficacy of WT-RBD-immunized mouse serum against SARS-CoV-2 WT and Omicron subvariants. (c–h), Synergism of EK1 and WT-RBD-immunized mouse sera against WT SARS-CoV-2 (c) BA.2.12.1 (d) BA.4/BA.5 (e) BA.4.6 (f) BA.2.75 (g) and BF.7 (h). Samples were tested in triplicate, and the experiment was repeated once. Data from a representative experiment are presented in mean ± SD. Asterisks indicate significant differences (***P < 0.001, **P < 0.01).

Currently, more than 12 billion vaccine doses have been administered globally [31], including inactivated vaccines, mRNA-based vaccines and vector-based vaccines, most of which are based on WT SARS-CoV-2 sequences. Therefore, those vaccines elicited high titers of WT RBD neutralizing antibodies [25]. However, as consistently shown in this report, a large number of RBD mutations enable Omicron subvariants to evade the neutralizing efficacy of WT RBD-specific antibodies and escape immune surveillance in people immunized with COVID-19 vaccines. Indeed, while we found that sera from WT-RBD-immunized mice (1:500 dilution) could significantly inhibit WT SARS-CoV-2 infection, little efficacy was shown against Omicron subvariants, including BA.2.12.1, BA.2.75, BA.4/BA.5, BA.4.6, and BF.7 (Figure 4(A,B)).

Since both RBD and HR1 are key in mediating viral entry [22], it is reasonable to speculate about the potential efficacy resulting from the synergism between HR1-based fusion inhibitors and WT-RBD-immunized mouse sera. Therefore, we herein used EK1 as a representative fusion inhibitor, which is currently in phase I/II clinical stage, to further explore its potential synergistic effect when combined with SARS-CoV-2 vaccine-induced antibodies. As shown in Figure 2(C), WT-RBD-immunized mouse sera potently inhibited WT (D614G) SARS-CoV-2 infection with 94.1% inhibition. On the other hand, low-dose EK1 (100 nM) only showed weak inhibition. However, when combined with RBD-immunized mouse sera, inhibition rose to 96.3%.

More importantly, WT-RBD-immunized mouse sera showed weak inhibitory activity against BA.2.12.1 and BA.2.75 with 31.4% and 33.1% inhibition, respectively, as did EK1 (100 nM) with 33.2% and 30.6% inhibition, respectively. However, when combined, they showed increased efficacy to 62.3% inhibition on BA.2.12.1 and 63% inhibition against BA.2.75 (Figure 4(D,E)). Similarly, WT-RBD-immunized mouse sera alone showed very limited inhibitory activity against BA.4/BA.5, BA.4.6, and BF.7 with 24.1% to 27.6% inhibition; however, after combining it with low-dose EK1 (100 nM), the new formulation showed potent synergism against BA.4/BA.5 with 55.1% inhibition, BA.4.6 with 55.5% inhibition and BF.7 with 54.7% inhibition (Figure 4(F–H)). Therefore, given the widespread coverage of SARS-CoV-2 vaccines, EK1-based fusion inhibitors could be good candidates for clinical application to combat the current Omicron subvariants with high resistance to neutralizing antibodies in vaccinees.

Consistently, BA2-convalescent sera (1:500 dilution) alone showed moderate inhibitory activity against the BA.2.12.1 sublineage with 64.6% inhibition, but little efficacy against BA.2.75, BA.4/BA.5, BA.4.6, BF.7, and WT SARS-CoV-2 (Figure S6). However, BA2-convalescent sera, when combined with low-dose EK1 (100 nM), showed significantly improved efficacy against BA.2.12.1, BA.2.75, BA.4/BA.5, BA.4.6, and BF.7 in the range of 57.9% to 83.0% inhibition (Figure 4(I–K) and Figure S6), further suggesting that EK1-based fusion inhibitors could effectively block viral reinfections of future emerging Omicron subvariants.

## Conclusions

Viral fusogenicity, an apparent determinant of viral pathogenicity, is widely concerning [19,32]. Our results suggest that Omicron BA.1 exhibited significantly decreased fusogenicity on target cells either bearing human ACE2 or horse-ACE2, cattle-ACE2, swine-ACE2, rabbit-ACE2, civet-ACE2 or bat-ACE2 receptors, compared to WT SARS-CoV-2. However, compared to the ancestral Omicron strain, we found that these recently emerging Omicron subvariants showed significantly increased capacity to mediate membrane fusion, especially BA.4/BA.5, BA.2.75, BA4.6, and BF.7, which are the currently dominant subvariants [33], indicating that improved viral fusogenicity might be an important evolutionary direction for the current Omicron subvariants and, as such, should be closely monitored. Meanwhile, we found that our EK1-based pan-CoV fusion inhibitor is equally effective against membrane fusion mediated by the S proteins of these Omicron subvariants and their PsV entry, as well as WT or Delta PsV. In our previous study, EK1 peptide exhibited low immunogenicity as no anti-EK1 antibodies were detected in mice 2 weeks after intranasal administration once daily for one week [15]. More importantly, we found that the combination of EK1 and WT-RBD-immunized mouse sera or BA2-convalescent sera showed potent synergism against multiple Omicron subvariants, including BA.2.12.1, BA.2.75, BA4/BA.5, BA.4.6, and BF.7. Given the widespread vaccination background of WT-SARS-CoV-2-based vaccines, as well as the long-term epidemic history of various SARS-CoV-2 subvariants, EK1-based pan-CoV fusion inhibitors show promise for combating the current Omicron epidemic.

## Methods

### Cells, serum sample and plasmids

The 293T cells were from ATCC (Manassas, VA, USA); HeLa and Caco2 cell lines were from the Chinese Academy of Science Cell Bank (Shanghai, China). 293T/ACE2 cells were preserved in our laboratory. All cell lines were cultured in Dulbecco’s Modified Eagle’s Medium (DMEM) with 10% fetal bovine serum (FBS). Plasmids, including pAAV-SARS-CoV-2-S-D614G-IRES-EGFP, pAAV-SARS-CoV-2-S-Delta-IRES-EGFP, pAAV-SARS-CoV-2-S-Omicron-IRES-EGFP, and PC-hACE2/horse_ACE2/cattle_ACE2/swine_ACE2/rabbit_ACE2/civet_ACE2/ bat_ACE2, were synthesized or preserved in our laboratory. Peripheral blood samples were collected from a convalescent BA.2 patient. Serum sample was isolated from centrifuged blood sample for inhibiting pseudovirus infection with 1:500 dilution. All collections were conducted according to the guidelines of the Declaration of Helsinki and approved by the Institutional Review Board of the Ethics Committee of Shanghai Fourth People's Hospital (2022095-001).

### Mouse vaccination

Briefly, WT SARS-CoV-2 RBD-Fc (5 µg) formulated with an equal volume of Imject Alum adjuvant (Thermo Scientific) was used to vaccinate Balb/c mice (six-week-old) three times at two-week intervals, as previously described [34,35]. At the 48th day, sera were isolated from blood samples to inhibit SARS-CoV-2 pseudovirus infection (1:500 dilution). All animal experiments were performed according to protocols approved by the Institutional Laboratory of Animal Care of Fudan University (20210302-083).

### Western blot

Western blot was performed using an anti-SARS-CoV-2 S antibody and an anti-actin antibody, as previously described [36]. Briefly, after transfection for 36 h, effector cells bearing S protein on their surface were collected. Samples were prepared to run an SDS-PAGE in 10% gels (Bio-Rad, Hercules, CA) and then transfer onto PVDF membranes. Membranes were blocked with 5% BSA in PBST for 2 h, followed by incubation with a SARS-CoV-2 S antibody (Sino Biological Inc., Beijing, China, Cat: 40592-T62), or a beta-actin mouse McAb (ProteinTech, Manchester, UK, Cat: 66009-1-Ig), for another 2 h at room temperature. Horseradish peroxidase (HRP)-conjugated polyclonal Goat anti-Rabbit IgG (1:5000) (DAKO, Cat: P0448) and Goat anti-Mouse IgG (1:5000) (Abcam, Cambridge, UK, Cat: ab6789) were used as secondary antibodies. Proteins were visualized using one-step ECL substrates (Meilunbio, Dalian, China).

### Cell–cell fusion assays

Plasmid pAAV-IRES-S-EGFP, encoding S protein and EGFP, was transfected into 293T effector cells (293T/S/GFP). Caco2 cells, naturally expressing human ACE2 receptors on the membrane surface, were used as target cells. 293T cells, transfected with plasmid pAAV-IRES-EGFP (293T/EGFP), were used as negative control. Effector cells (293T/S/GFP) were collected and resuspended. Free effector cells were added into target cells (Caco2 or HeLa cells expressing proteins of hACE2, horse_ACE2, cattle_ACE2, swine_ACE2, rabbit_ACE2, civet_ACE2, or bat_ACE2, respectively) for coincubation for indicated time at 37 °C and then observation under the fluorescence microscope.

### Inhibition of pseudotyped virus infection

HIV-1 backbone-based Pseudovirus (PsV) bearing wild or mutant SARS-CoV2 S protein was produced, as previously described [27]. Caco2 cells were used as target cells and were seeded in wells of a 96-well plate 48 h in advance. PsV in the presence or absence of each peptide at indicated concentrations was added into target cells. After 12 h, culture medium was refreshed and continuously cultured for an additional 48 h. Then, cells were lysed with lysis reagent (Promega) for relative light units (RLU) detection by using Luciferase Assay Kits.

### Statistical analyses

Statistical analyses were carried out using GraphPad Prism 8.0. Analyses of independent data were carried out through Student’s unpaired two-tailed *t*-test and ANOVA test. *P* values less than 0.05 were significant; **P < 0.01; ***P < 0.001. The concentration for half inhibition (IC50) was calculated by CalcuSyn software [37].

## Supplementary Material

Supplemental Material
