# Supplementary material for: SARS-CoV-2 Omicron subvariants exhibit distinct fusogenicity, but similar sensitivity, to pan-CoV fusion inhibitors
Source: Emerg Microbes Infect. 2023 Feb 23;12(1):2178241. doi: 10.1080/22221751.2023.2178241 (PMC9970205; doi:10.1080/22221751.2023.2178241)
Supplement: Supplemental Material [file TEMI_A_2178241_SM9022.docx]

Supplementary Material for

**SARS-CoV-2 Omicron subvariants exhibit distinct fusogenicity, but similar sensitivity, to pan-CoV fusion inhibitors**

Shuai Xia^a^*†, Lijue Wang ^a^*, Fanke Jiao ^a^*, Xueying Yu ^b^, Wei Xu ^a^, Ziqi Huang, Xicheng Li, Qian Wang ^a^, Yun Zhu^c^, Qiuhong Man ^b^†, Shibo Jiang ^a^†, Lu Lu ^a^†

**Affiliations:**

*^a^Key Laboratory of Medical Molecular Virology (MOE/NHC/CAMS), Shanghai Institute of Infectious Disease and Biosecurity, School of Basic Medical Sciences, Shanghai Frontiers Science Center of Pathogenic Microbes and Infection, Fudan University, Shanghai, China.*

*^b^Department of Clinical Laboratory, Shanghai Fourth People's Hospital, School of Medicine, Tongji University, Shanghai, China.*

*^c^National Laboratory of Biomacromolecules, Institute of Biophysics, Chinese Academy of Sciences, Beijing, China*

*These authors contributed equally to this work.

†Corresponding author. Email: lul@fudan.edu.cn (L.L.); [shibojiang@fudan.edu.cn](mailto:shibojiang@fudan.edu.cn) (S.J.); [Manqiuhong307@163.com](mailto:Manqiuhong307@163.com) (M.Q.); sxia15@fudan.edu.cn (S.X.).

**This file includes:**

**Supplementary Fig. 1. Schematic representation of SARS-CoV-2 WT(D614G)-S, Delta-S, Omicron-S proteins and their mutants.**

**Supplementary Fig. 2. Fusogenicity of WT and Omicron-BA1 S proteins on HeLa cells expressing horse-ACE2 (a), cattle-ACE2 (b), swine-ACE2 (c), rabbit-ACE2 (d), civet-ACE2 (e) or bat-ACE2 (f) receptors.**

**Supplementary Fig. 3. Fusion rates mediated by SARS-CoV-2 WT-S and Omicron-S proteins after coculture for 2 hours.**

**Supplementary Fig. 4.** **The interaction of SARS-CoV-2 HR1 domain with EK1 peptide.**

**Supplementary Fig. 5. Inhibition of EK1 and EK1C4 against WT-S-mediated cell-cell fusion (a) and pseudovirus infection (b).**

**Supplementary Fig. 6. Potent synergism of EK1 combined with BA2-convalescent sera against Omicron subvariants.**


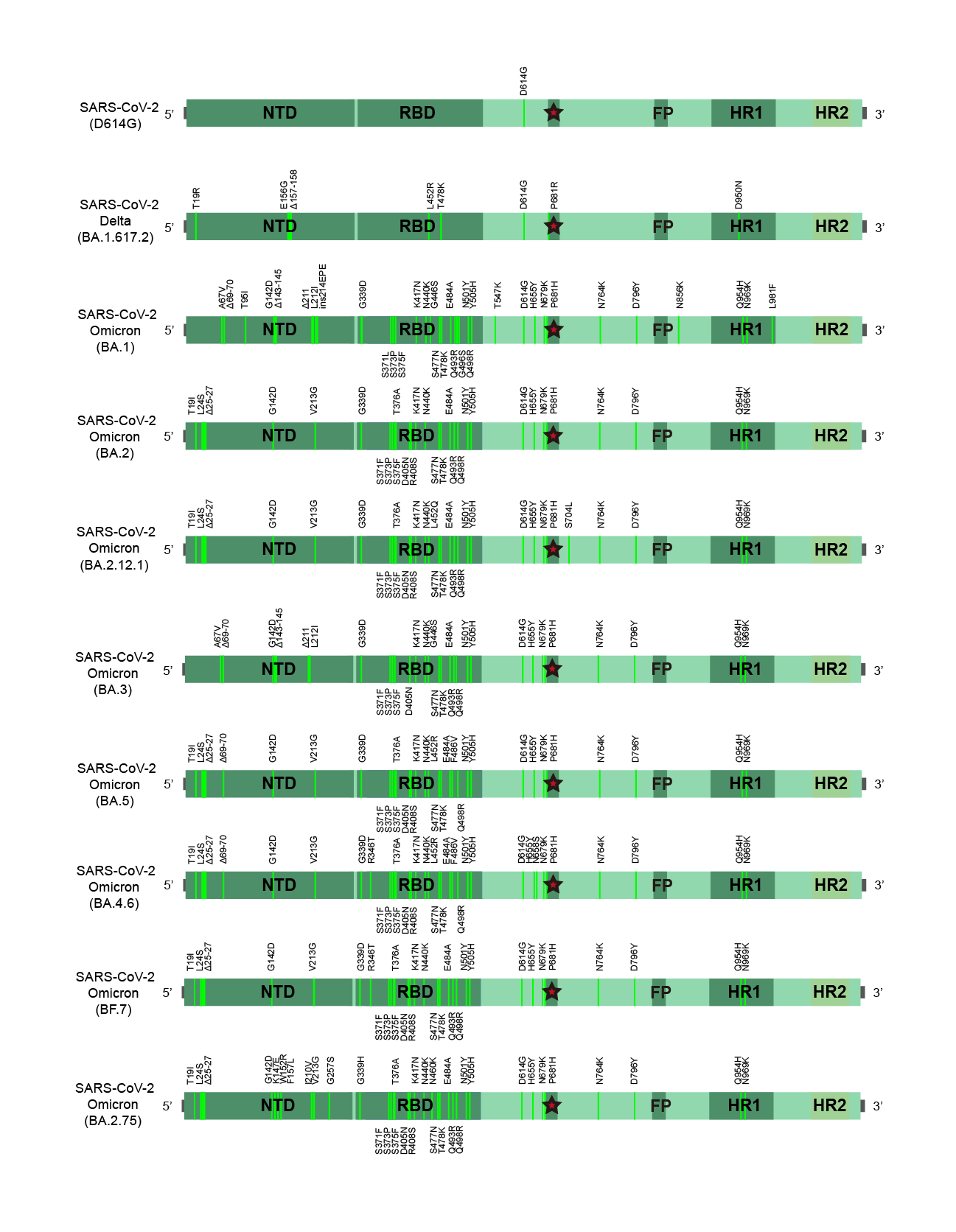


**Supplementary Fig. 1. Schematic representation of SARS-CoV-2 WT(D614G)-S, Delta-S, Omicron-S proteins and their mutants.**

**
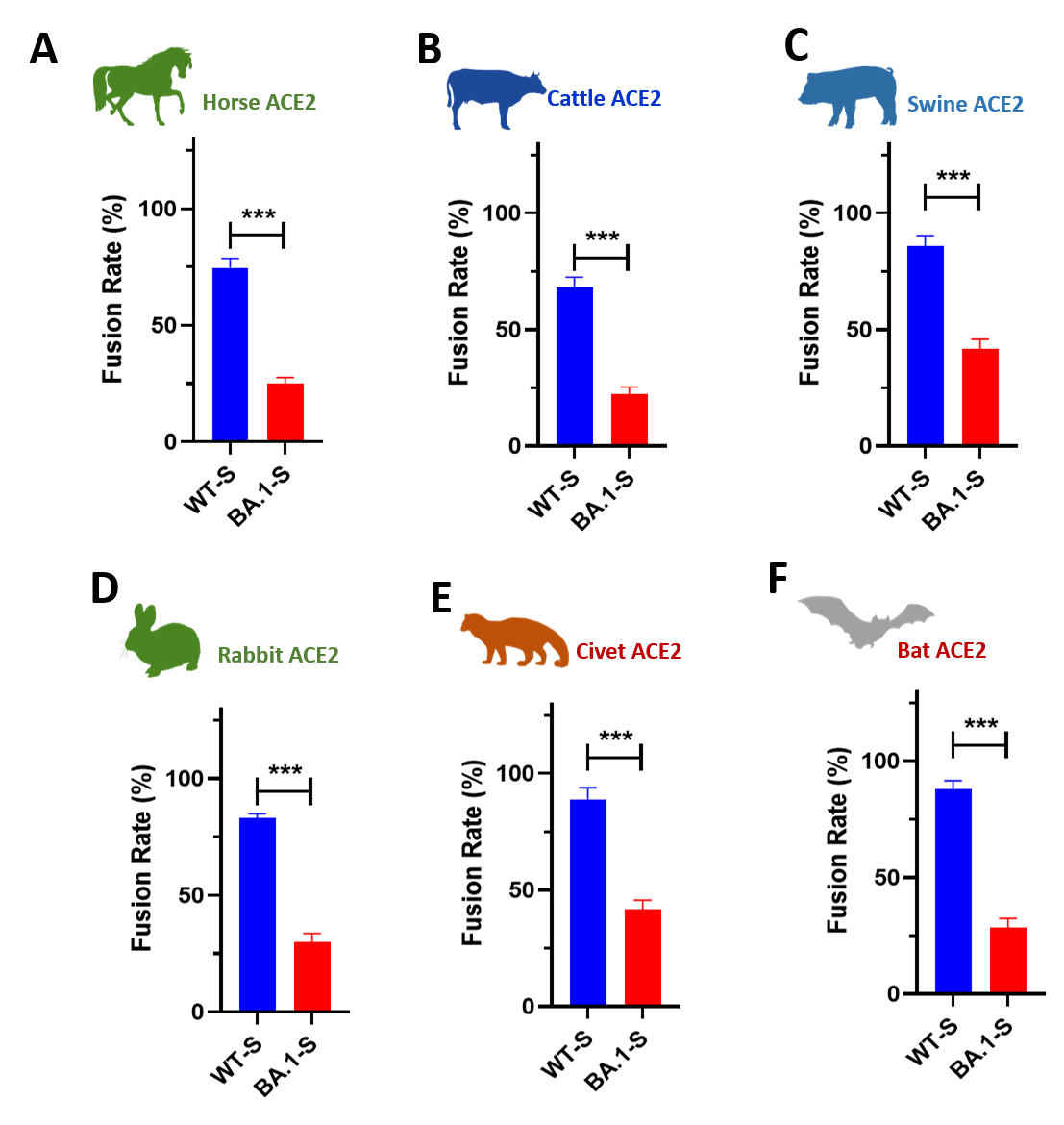
**

**Supplementary Fig. 2. Fusogenicity of WT and Omicron-BA1 S proteins on HeLa cells expressing horse-ACE2 (a), cattle-ACE2 (b), swine-ACE2 (c), rabbit-ACE2 (d), civet-ACE2 (e) or bat-ACE2 (f) receptors.**


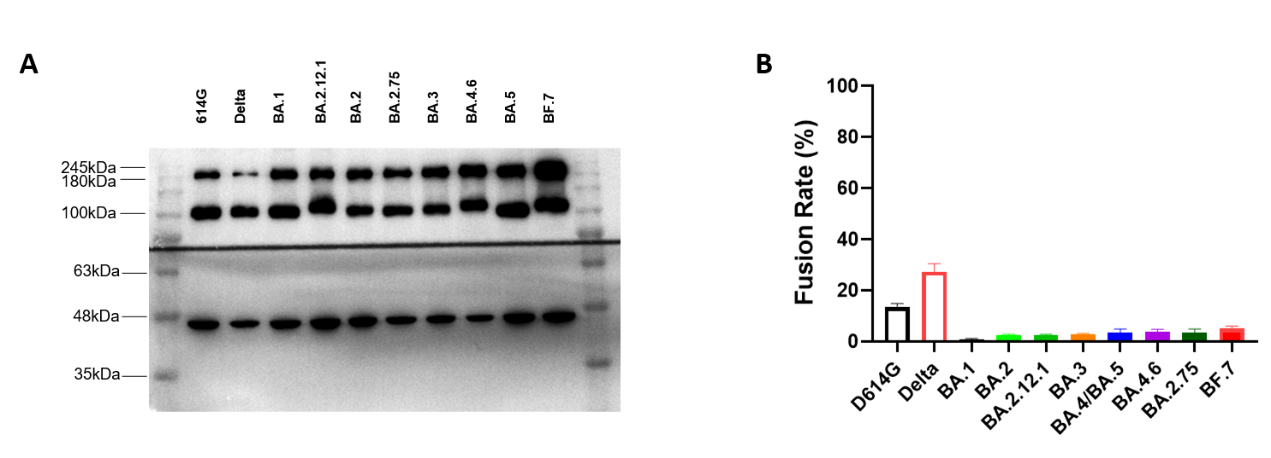


**Supplementary Fig. 3. Fusion rates mediated by SARS-CoV-2 WT-S and Omicron-S proteins after coculture for 2 hours.** (a), Western blot analysis of S protein expression in effector cells. (b), Statistical analysis of fusion rates mediated by SARS-CoV-2 WT-S and Omicron-S proteins after coculture for 2 hours on Caco2 cells.

**
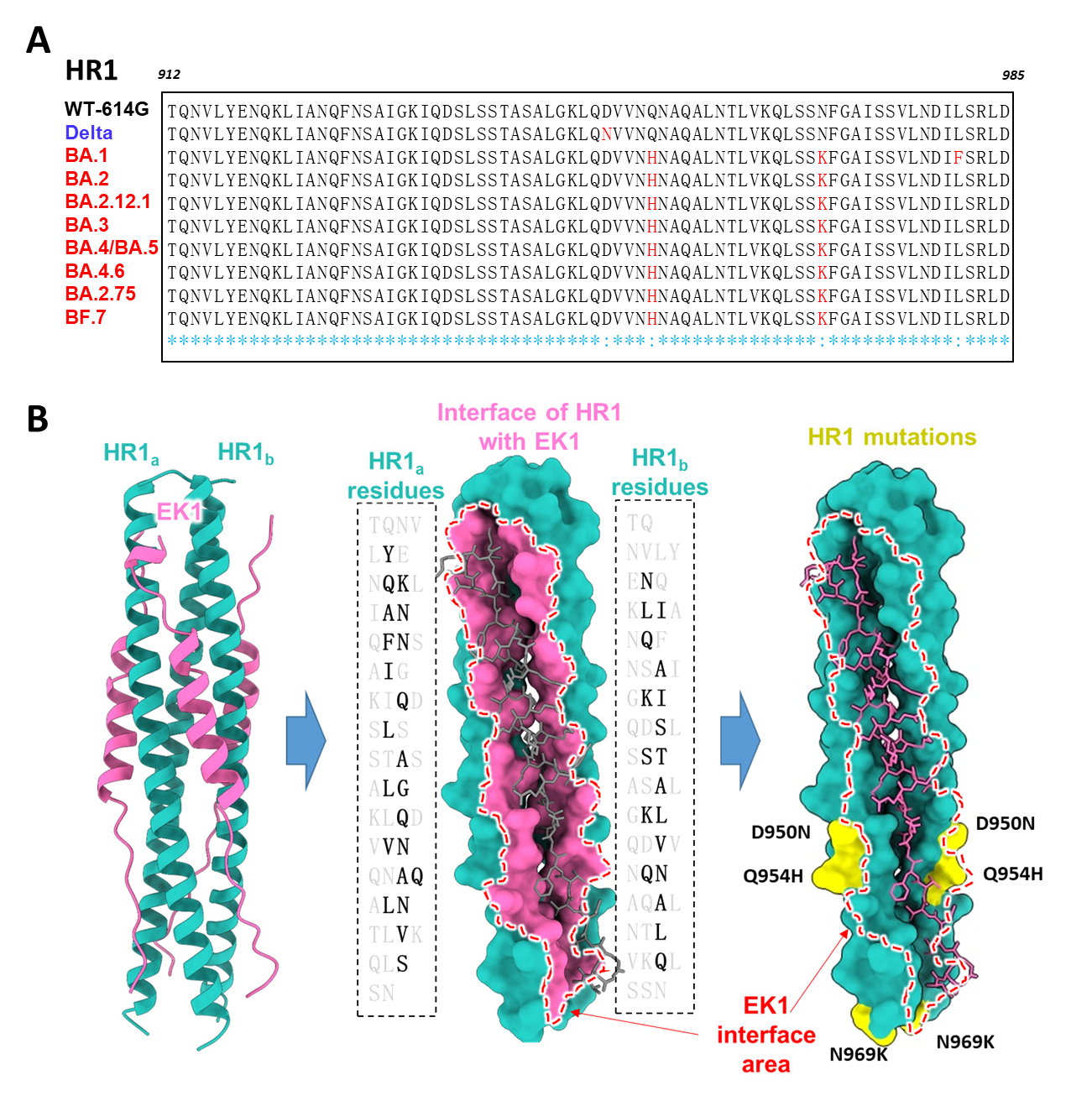
**

**Figure S4. The interaction of SARS-CoV-2 HR1 domain with EK1 peptide.** (a), Sequence alignment of HR1 domains in WT-S, Delta-S and Omicron-S. (b), The interaction interface of SARS-CoV-2 HR1 domain with EK1 peptide. In the left panel, the crystal structure of HR1-EK1 complex (PDB entry 7C53) are shown in cartoon representation. The HR1 protomers are coloured light sea green, and the EK1 protomers are coloured hot pink. In the middle panel, two HR1 protomers are shown in surface representation, and their interfaces with EK1 are colored in hot pink. The EK1 protomer is shown in sticks representation, and EK1-binding residues of HR1 are shown with bold black letter. The boundaries of the interface area are indicated by red dashed lines. In the right panel, the point mutations in HR1 domain are coloured yellow on the HR1 surface and indicated.

**
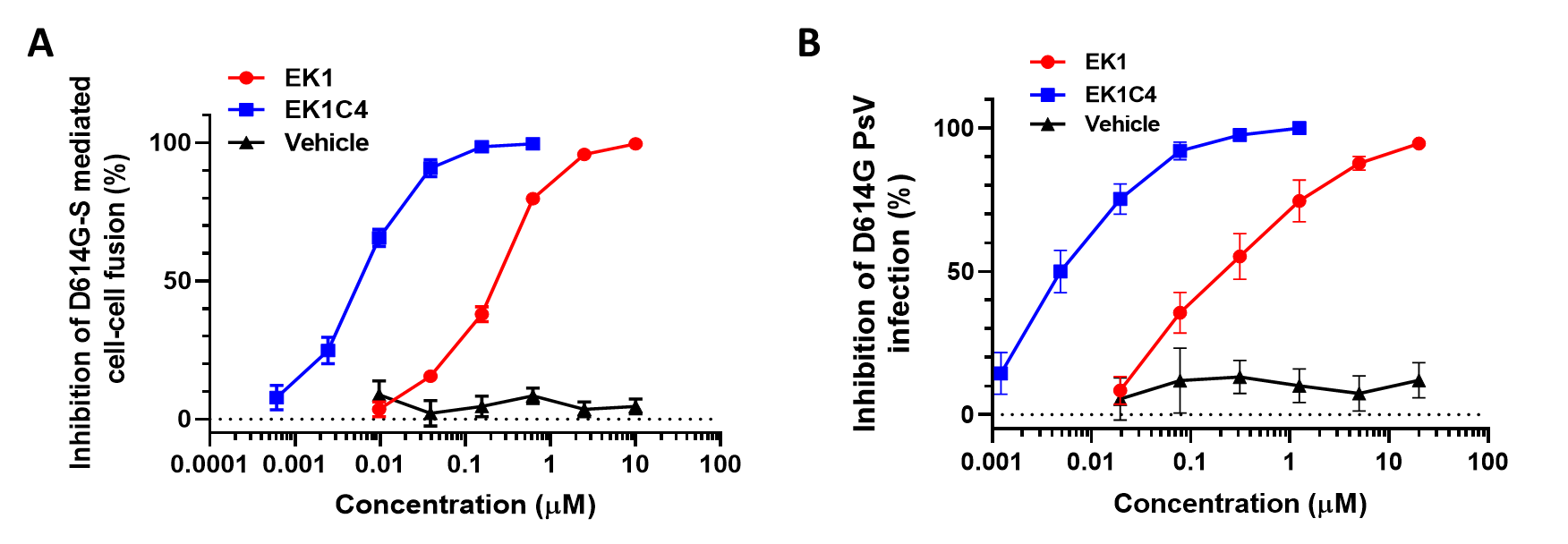
**

**Supplementary Fig. 5.** **Inhibition of EK1 and EK1C4 against WT-S-mediated cell-cell fusion (a) and pseudovirus infection (b).**

**
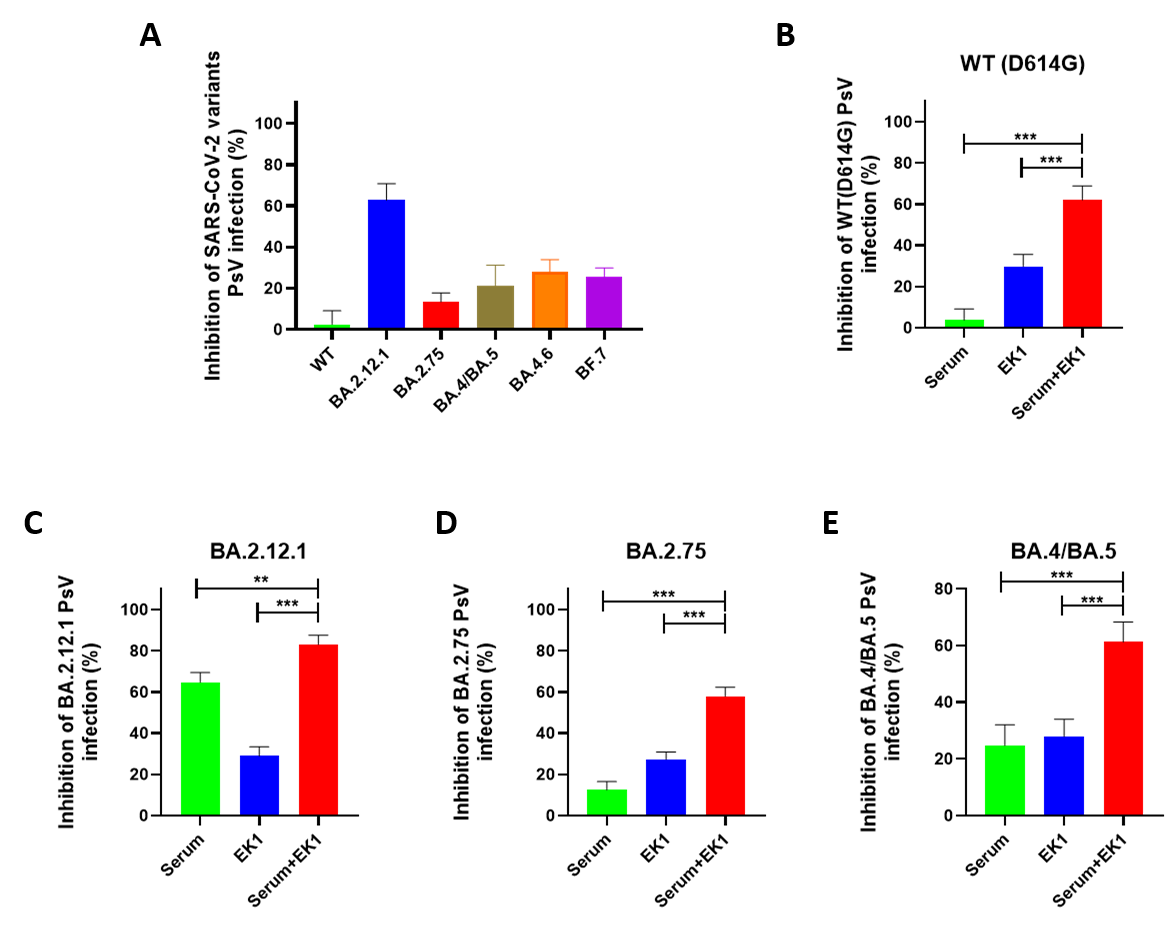
**

**Supplementary Fig. 6. Potent synergism of EK1 combined with BA2-convalescent sera against Omicron subvariants.**
